# Editorial: Neuronal guidance signaling in health and neurological diseases

**DOI:** 10.3389/fcell.2025.1661836

**Published:** 2025-07-18

**Authors:** Satoru Yamagishi, Masaki Hiramoto, Junichi Yuasa-Kawada

**Affiliations:** ^1^Department of Optical Neuroanatomy, Institute of Photonics Medicine, Hamamatsu University School of Medicine, Hamamatsu, Japan; ^2^Department of Molecular and Cellular Biology, The Scripps Research Institute, La Jolla, CA, United States; ^3^Department of Neurology, Juntendo University Faculty of Medicine, Tokyo, Japan

**Keywords:** axon guidance, brain development, guidance signaling, neuropsychiatric disorders, neurodegenerative disorders

Neuronal guidance signaling represents a cornerstone found in neuroscience, vital for the precise establishment of neural circuits during development. Neurons navigate complex environments, extending axons to target locations and forming synaptic connections through the interpretation of diverse extracellular cues. Central to this intricate process are neuronal guidance genes, encoding proteins that act as cues, receptors, or intracellular signaling effectors. These molecular players ensure accurate neural wiring, synapse formation, and ongoing neural maintenance throughout life (Yamagishi et al., 2021; Yuasa-Kawada et al., 2023). Disruption or dysregulation in these signaling pathways underlies many developmental, neuropsychiatric, and neurodegenerative disorders (Yuasa-Kawada et al., 2026). This Research Topic compiles original research and insightful reviews aimed at exploring both novel and classical mechanisms underlying neuronal guidance signaling, highlighting significant progress and identifying critical areas for future exploration to open new avenues toward developing clinical applications.

One of key signaling molecule extensively studied is Draxin, an axon guidance protein essential for the development of forebrain commissures. Shinmyo reviews the role of draxin, emphasizing its involvement in neurological disorders such as autism spectrum disorder (ASD). Draxin knockout mice display significant structural anomalies, notably in the corpus callosum, hippocampal commissure, and thalamocortical projections. Interestingly, the deletion of draxin gene was identified in ASD model BTBR/J mice, suggesting that draxin deletion is a genetic factor for ASD-like characteristics in the mice. Genetic manipulations further support draxin’s essential function in establishing neural circuitry and highlight its potential role as a genetic determinant of ASD-related neuroanatomical changes.

Moving from axon guidance proteins to transcriptional regulation, Tsuboi and Yoshihara reviewed the Aristaless-related homeobox (Arx) gene. Arx mutations have been linked with a variety of neurological disorders, including intellectual disability and epilepsy. Their review emphasizes the pivotal role of Arx in the development and migration of GABAergic interneurons, especially within the cerebral cortex and olfactory bulb. By employing conditional knockout strategies, recent findings have identified Arx as a crucial regulator of interneuron progenitor differentiation, highlighting its importance in neurodevelopmental disorders and cortical interneuron specification.

Nickerson et al. provide new insights into the complexities of axon guidance at the spinal cord midline through their study on Slit-Robo signaling. Utilizing genetic mouse models, they uncover that Robo receptors counteract DCC-mediated attraction to Netrin-1, preventing motor neurons and their axons from aberrant midline crossing. Their work reveals a sophisticated interplay between Slit and netrin signaling pathways, demonstrating how Slit proteins convert netrin’s attractive cues into repulsive signals, a mechanism critical for precise motor circuit formation.

Complementing this, Northington et al. focus on the molecular interplay downstream of netrin-1 signaling, specifically how microtubule modifications mediate guidance responses. Their findings indicate that the polyglutamylase enzyme TTLL1 is required for netrin-1-induced axon growth, highlighting a previously underappreciated layer of complexity involving post-translational modifications of microtubules in guidance signaling. This discovery not only deepens our understanding of cytoskeletal dynamics in axon guidance but also offers new avenues for exploring therapeutic targets for conditions involving disrupted axonal pathfinding.

Exploring further downstream signaling mechanisms, Hale and Bashaw discuss the emergent roles of E3 ubiquitin ligases in neural development. Their review covers how ubiquitination, mediated by specific E3 ligases, regulates protein localization, degradation, and signaling. By examining ligase families such as RING and HECT, they elucidate their roles in neural specification, axon guidance, and dendrite morphogenesis. Critically, these ligases are linked to various neurodevelopmental disorders, emphasizing their potential as therapeutic targets to manage conditions like autism spectrum disorder, Angelman syndrome, and intellectual disability.

A novel intersection between bone-derived hormones and neural signaling is addressed by Bian et al., who discuss osteocalcin (OCN) and its receptor GPR37. Osteocalcin, traditionally associated with bone metabolism, has been recognized as an endocrine regulator influencing cognitive function and mood. GPR37, prominently expressed in the brain, mediates osteocalcin signaling, affecting neuronal migration, proliferation, and differentiation. This receptor pathway has potent neuroprotective effects, implicating it in neurodegenerative diseases like Parkinson’s disease, and offering a unique perspective on the bone-brain axis in neurological health.

In parallel, Li et al. expand on osteocalcin signaling, examining GPR158, another key receptor involved in CNS functions. They propose that GPR158 plays a critical role in synaptic plasticity and cognition, influencing stress responses and metabolic regulation. The receptor is intricately linked with neurodegenerative diseases, suggesting that further exploration could yield valuable therapeutic interventions targeting cognitive and mood disorders through modulation of the bone-brain endocrine axis.

Further enriching this Research Topic, Atkins et al. address the novel perspective of primary cilia in neuronal guidance. Traditionally considered vestigial, primary cilia are now recognized as critical signaling antennas that concentrate neuronal guidance receptors. This review emphasizes the necessity of future investigations into ciliary signaling pathways, given their emerging relevance in neurological diseases including ciliopathies, neurodevelopmental, and neurodegenerative disorders.

Finally, Nguyen et al. provide critical perspectives on the role of LRRK2, leucine-rich repeat kinase 2, widely known for its association with Parkinson’s disease. They underscore recent findings suggesting that LRRK2 and its orthologs not only prevent neurodegeneration but also safeguard against developmental defects, notably influencing axon guidance. Their discussion highlights autophagy regulation as a key pathway, indicating how disruptions in LRRK2 functions can simultaneously lead to neurodevelopmental abnormalities and later-life neurodegenerative conditions.

Collectively, these contributions underscore how neuronal guidance signaling extends beyond developmental contexts, implicating diverse signaling pathways and molecular mechanisms in lifelong neural health and disease. Advancements in understanding these intricate interactions not only enrich basic neuroscience but also pave the way for developing innovative therapeutic approaches to neurodevelopmental and neurodegenerative diseases. As this Research Topic demonstrates, exploring neuronal guidance signaling continues to reveal unexpected molecular players and mechanisms that could revolutionize our approach to neurological healthcare. This editorial emphasizes the interconnectedness of neuronal signaling pathways, urging continued collaborative exploration across disciplinary boundaries to fully uncover the complexities of brain development and pathology.
